# Binocular rivalry in children on the autism spectrum

**DOI:** 10.1002/aur.1749

**Published:** 2017-03-16

**Authors:** Themelis Karaminis, Claudia Lunghi, Louise Neil, David Burr, Elizabeth Pellicano

**Affiliations:** ^1^ Centre for Research in Autism and Education, Department of Psychology and Human DevelopmentUCL Institute of Education, University College LondonLondonUnited Kingdom; ^2^ School of PsychologyPlymouth UniversityPlymouthUnited Kingdom; ^3^ Department of Translational Research and New Technologies in Medicine and SurgeryUniversity of PisaPisaItaly; ^4^ Institute of Neuroscience, National Research Council (CNR)PisaItaly; ^5^ Department of Neuroscience, Psychology, Pharmacology and Child HealthUniversity of FlorenceFlorenceItaly; ^6^ School of Psychological ScienceThe University of Western AustraliaCrawleyAustralia

**Keywords:** binocular rivalry, autism, perception, bistable perception, vision, atypical development

## Abstract

When different images are presented to the eyes, the brain is faced with ambiguity, causing perceptual bistability: visual perception continuously alternates between the monocular images, a phenomenon called binocular rivalry. Many models of rivalry suggest that its temporal dynamics depend on mutual inhibition among neurons representing competing images. These models predict that rivalry should be different in autism, which has been proposed to present an atypical ratio of excitation and inhibition [the E/I imbalance hypothesis; Rubenstein & Merzenich, 2003]. In line with this prediction, some recent studies have provided evidence for atypical binocular rivalry dynamics in autistic adults. In this study, we examined if these findings generalize to autistic children. We developed a child‐friendly binocular rivalry paradigm, which included two types of stimuli, low‐ and high‐complexity, and compared rivalry dynamics in groups of autistic and age‐ and intellectual ability‐matched typical children. Unexpectedly, the two groups of children presented the same number of perceptual transitions and the same mean phase durations (times perceiving one of the two stimuli). Yet autistic children reported mixed percepts for a shorter proportion of time (a difference which was in the opposite direction to previous adult studies), while elevated autistic symptomatology was associated with shorter mixed perception periods. Rivalry in the two groups was affected similarly by stimulus type, and consistent with previous findings. Our results suggest that rivalry dynamics are differentially affected in adults and developing autistic children and could be accounted for by hierarchical models of binocular rivalry, including both inhibition and top‐down influences. ***Autism Res*** *2017*. ©2017 The Authors Autism Research published by Wiley Periodicals, Inc. on behalf of International Society for Autism Research ***Autism Res** 2017, 10: 1096–1106*. © 2017 International Society for Autism Research, Wiley Periodicals, Inc.

## Introduction

The phenomenon of binocular rivalry offers useful insights into the neural dynamics of perception [e.g., Blake, Brascamp, & Heeger, 2014]. Binocular rivalry occurs when the two eyes are presented with incompatible images, which cannot be fused into a single percept [Breese, 1909; Levelt, 1965; Wheatstone, 1838; Whittle, 1965; for more recent reviews, see Alais & Blake, 2005; Howard & Rogers, 2012]. Under these dichoptic stimulation conditions, visual perception oscillates between the two monocular images every few seconds, as the observer attempts to interpret the visual world given inconsistent and competing information. At the time of perceptual transitions, periods of mixed perception occur during which viewers observe an amalgamation of the two images.

One important question is how the brain resolves the competition between the two mutually exclusive percepts. Many models of binocular rivalry [e.g., Blake, Yu, Lokey, & Norman, 1998; Freeman, 2005; Lehky, 1988; Noest, van Ee, Nijs, & van Wezel, 2007; Wilson, 2003] suggest that this happens through lateral inhibition between monocular neurons. Mutual inhibition between groups of neurons representing the competing images results in the suppression of the weaker percept and the build‐up of the stronger one. Inhibition also interacts with neuronal adaptation and neuronal fatigue and this interplay could underlie perceptual switches [Alais, Cass, O'Shea & Blake, 2010; Blake, 1989; Platonov & Goossens, 2013]. Experimental evidence for the involvement of inhibition in binocular rivalry has been provided by MR Spectroscopy studies, which have demonstrated a strong relationship between GABA concentration in the primary human visual cortex and rivalry dynamics [Lunghi, Emir, Morrone, & Bridge, 2015; van Loon et al., 2013].

There is also alternative evidence [animal model neurophysiology: Jamain et al., 2008; Markram, Rinaldi, & Markram, 2007; epidemiological: Levisohn, 2007] that suggests that cortical inhibition is atypical in autism, most likely due to differences in the release or the signaling of excitatory (glutamate) and inhibitory (GABA) neurotransmitters. Based on these findings, the excitation/inhibition (E/I) imbalance hypothesis of autism [Rubenstein & Merzenich, 2003; Vattikuti & Chow, 2010] proposes that a wide range of sensory, as well as language and social symptoms of autistic individuals might be due to an atypical proportion of excitation and inhibition in the related brain systems.

The E/I hypothesis is also particularly relevant to binocular rivalry. As inhibition is a key process in the phenomenon [Blake et al., 1998; Freeman, 2005; Lehky, 1988; Lunghi et al., 2015; Noest et al., 2007; van Loon et al., 2013; Wilson, 2003], an altered E/I ratio should result in atypical rivalry dynamics in autistic individuals. Intriguingly, two initial studies evaluating this prediction produced contrary results. Said, Egan, Minshew, Behrmann, and Heeger [2013] measured binocular rivalry between orthogonal gratings in groups of autistic and typical adults and found no group differences in mixed percepts duration and in traveling wave dynamics during binocular rivalry. However, Robertson, Kravitz, Freyberg, Baron‐Cohen and Baker [2013], measuring binocular rivalry between complex visual stimuli (object images), found that autistic adults presented slower alternation rate and longer durations of mixed percepts compared to typical adults. Furthermore, both of these parameters of binocular rivalry were also highly predictive of clinical measures of autistic symptomatology [ADOS‐G; Lord, Rutter, DiLavore, & Risi, 1999]. Notably, however, more than half of the autistic participants failed to reach the cut‐off for an autism spectrum disorder on the diagnostic assessment, the ADOS‐G [but see Freyberg, Robertson, & Baron‐Cohen, 2015, Robertson, Ratai, & Kanwisher, 2016].

Robertson et al.'s [2013] results have recently been replicated [Freyberg et al., 2015; Robertson et al., 2016]. Freyberg et al. [2015] measured binocular rivalry between two types of stimuli, gratings [low complexity, as in Said et al., 2013] and objects [high complexity/nonsocial, as in Robertson et al., 2013], and tested a slightly larger sample of adult participants, which was also more rigorously assessed for autistic symptomatology. This study also showed that the altered rivalry dynamics found in the autistic group did not depend on stimulus complexity: mixed‐percept durations were longer for gratings than for objects in both groups.

Robertson et al. [2016] recently found slower alternation rates and lower proportions of dominant percepts in autistic compared to neurotypical adults tested on the rivalry stimuli of Robertson et al. [2013]. The new study also looked at the relationship between the levels of neurotransmitters GABA and glutamate, measured with MR Spectroscopy, in the visual cortex and rivalry dynamics. The levels of GABA strongly predicted rivalry dynamics in typical adults. Yet, strikingly, no such relationship was found for autistic participants. Unlike GABA, glutamate presented a similar link with rivalry dynamics in the two groups. As the authors discuss, these combined findings suggested a link between excitation/inhibition imbalance and a specific perceptual feature of autism.

All of the studies investigating binocular rivalry in autism thus far have focused on autistic adults. The developmental origins of atypical rivalry dynamics in autistic adults are therefore not yet known. In the current study, we address this issue directly by examining whether altered binocular rivalry dynamics are present earlier during development—in autistic children aged between 6 and 14 years. We developed a child‐friendly paradigm to measure binocular rivalry, which we administered to age‐ and ability‐matched groups of autistic and typical children. Similar to Freyberg et al. [2015], we included two levels of stimulus complexity, low (gratings) and high (houses/faces). The high complexity stimuli in our study also incorporated the contrast between socially‐relevant (faces) and nonsocial visual information (houses), as autistic children and adults often show atypicalities in the processing of socially‐relevant information [Uljarevic & Hamilton, 2012]. We therefore hypothesized that these atypicalities might translate into atypical rivalry dynamics in autistic children specifically for socially relevant stimuli.

## Methods

### Participants

Participants' demographics are shown in Table 1.

**Table 1:** Participant Demographics

**Measure**	**Children With Autism**	**Typically Developing Children**	**Statistical Comparison**
*N*	16	20	
Gender (*n* males: *n* females)	13: 3	10: 10	***χ*** ^2^(1, *N* = 36) = 3.76, *P* = 0.052
*Age* (Years)			
Mean SD)	9.9(2.4)	9.4(2.1)	*t*(34) = 0.57,
Range	7.2–14.7	6.6–12.8	*P* = 0.57
*Verbal IQ* ^a^			
Mean (SD)	102.4(15.9)	106.5(12)	*t*(34) = 0.89,
Range	76–129	89–128	*P* = 0.38
*Performance IQ* ^a^			
Mean (SD)	102.2(19.1)	98.7(13.4)	*t*(34) = 0.67,
Range	64–128	74–120	*P* = 0.51
*Full‐Scale IQ* ^a^			
Mean (SD)	102.9(17.8)	103 (12.1)	*t*(34) = 0.003,
Range	68–129	82–122	*P* = 0.99
*SCQ score* ^b^			
N	16	17	
Mean (SD)	19(7.4)	4(3.4)	*t*(31) = 7.59
Range	5–30	0–13	*P* < 0.001
*ADOS‐2 score* ^c^			
N	15	0	
Mean (SD)	9.4(2.4)	n/a	n/a
Range	7–15	n/a	

a aAs measured by the Wechsler Abbreviated Scales of Intelligence–2nd edition (WASI‐II; Wechsler, 2011).

b bSCQ: Social Communication Questionnaire [Rutter et al., 2003].

c cADOS‐2: Autism Diagnostic Observation Schedule–2nd edition [Lord et al., 2012]; higher scores on the SCQ and the ADOS‐2 reflect greater degrees of autistic symptomatology.

#### Autistic children

Sixteen autistic children (13 boys) aged between 7 and 14 years (*M* = 9.9; SD = 2.4) were recruited via schools in London and community contacts. All children had been previously diagnosed with an autism spectrum condition by independent clinicians. Children were administered the Autism Diagnostic Observation Schedule‐2nd Edition [ADOS‐2; Lord et al., 2012; *n* = 15] and parents also completed the Lifetime version of the Social Communication Questionnaire [SCQ; Rutter, Bailey, & Lord, 2003; *n* = 16], a screening test for autism (see Table 1 for scores). Children were included in data analysis if they had an independent clinical diagnosis of autism and scored above threshold for an autism spectrum disorder (ADOS‐2 cut‐off score = 7; SCQ cut‐off score = 15) in at least one of these two measures [Corsello et al., 2007].

#### Typically developing comparison children

Twenty typically developing children (10 boys), recruited from local London schools, were matched with autistic children for: chronological age, *t*(34) = 0.57, *P* = 0.57; verbal IQ, *t*(34) = 0.89, *P* = 0.38; performance IQ, *t*(34) = 0.67, *P* = 0.51; and full‐scale IQ, *t*(34) = 0.003, *P* = 0.99, as measured by the Wechsler Abbreviated Scales of Intelligence‐2nd edition [WASI‐II; Wechsler, 2011]–see Table 1. All children were considered to be cognitively able (verbal IQ, performance IQ and full‐scale IQ scores > = 70). There was a significant difference in gender, *χ* ^2^(1, *N* = 44) = 6.70, *P* = 0.009, with more girls in the typical group than in the autistic group. Parents of typical children also completed the SCQ (*n* = 17). Children's scores ranged between 0 and 13 (mean = 4, SD = 3.4), well below the cut‐off point for autism [score of 15; Rutter et al., 2003].

All children had normal or corrected‐to‐normal visual acuity, as reported by parents and as assessed using a Snellen acuity chart (binocular crowded‐letter acuity scores of 6/9 or better), and no strong eye‐dominance. Eye dominance was assessed as the ratio *dominant eye mean phase duration/non‐dominant eye mean phase duration* (see Data analysis).

Seven additional autistic children and eight additional typical children [same across groups, *χ* ^2^(1, *N* = 50) = 0.02, *P* = 0.88] took part in the study but were excluded from analyses because of strong eye dominance (eye dominance dominant/non‐dominant eye ratio > 2), leading to the final sample of 16 autistic and 20 typical children, as reported above. This was an important step: including children with strong eye dominance could induce serious artefacts to the data. For example, participants with increased eye dominance are likely to present lower switching rates, likely due to abnormal interocular competition resulting in one eye dominating for longer periods of time, rather than to a genuine difference in switching rate.

### Ethics Statement

This study was conducted in accordance to the principles laid down in the Declaration of Helsinki. Parents of all children gave their informed written consent prior to their child's participation in the project and children gave their verbal assent. The UCL Institute of Education's Faculty Research Ethics Committee approved all procedures (FPS456).

### Apparatus and Stimuli

**Figure 1:**
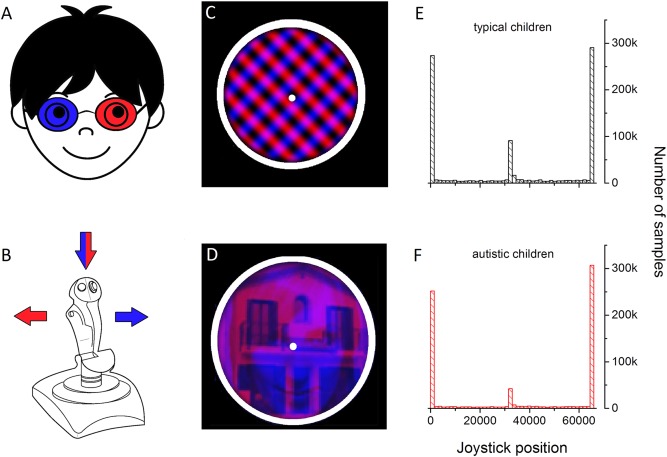
Schematic diagram of the visual stimuli and the experiment.

Visual stimuli were created in MATLAB (The Mathworks Inc., Natick, MA, USA) using PsychToolbox [Brainard, 1997; Kleiner, Brainard, & Pelli, 2007] and displayed on a 15‐inch monitor of a Dell Precision M4700 laptop driven at a resolution of 1366 x 768 pixels, at a refresh rate of 60 Hz. Participants viewed the monitor at a distance of 57 cm through anaglyph red‐blue goggles (right lens: blue, left lens: red). Responses were continuously recorded through a joystick (Fig. 1A). Visual stimuli were either two superimposed oblique orthogonal red and blue gratings (orientation: ±45°, size: 3°, SF 2 cpd or 3.5 cpd, RMS contrast 35%; Fig. 1B) or superimposed face and house separately defined by red and blue luminance variations (size 3°, MS RMS contrast 25%; Fig. 1C). The contrast energy of the house and face stimuli was equalized by calculating RMS contrast of the stimuli matrices (in grayscale) and changing the stimuli contrast until they were matched. To compensate for possible spectral differences when presenting the stimuli in different colors, each participant completed one experimental blocks in one color (e.g., red) and the second block in the other color (e.g., blue). The visual stimuli were surrounded by a white smoothed circle, presented on a black uniform background in central vision. Peak luminance of the red grating was matched with the physical peak luminance of the blue one (1.2 cd/m^2^, measured after passage through the goggle lenses).

### Task and Procedures

The task was presented in the context of a child‐friendly computer game, which included introduction, training and test phases.

#### Introduction phase

Children were told they were required to act as referees in a magic contest in which the magic tricks could only be seen through special goggles. As a referee, they were instructed to use the joystick to indicate which of two competing characters, one associated with the red color (“Fred Pepper”) and one with the blue color (“Berry Blue”), dominated at each time. Children were then shown how to hold the joystick to the left when they saw a red figure on screen; to the right when they saw the blue figure; and how to move/leave the joystick in the middle position when they saw a mixture of a red and a blue figure.

#### Training phase

This phase served to familiarize children with the task. It preceded each of the two blocks of the Test phase (see below) using the corresponding stimuli, that is, either gratings or houses/faces. During the training phase, children wore anaglyph goggles so as to mimic the actual binocular rivalry test. They were presented with a 30‐sec sequence of alternations between a red and a blue stimulus (either gratings or house/face), which were interleaved with transitions (50%‐50% mixes of the two images, created with OpenGL blending; Shreiner & OpenGL Architecture Review Board, 2006). The training phase therefore imitated the dynamics of binocular rivalry (mean durations of red, blue and mixed percepts = 2.30 ± 0.70 sec). The experimenter first guided the joystick with their hand, giving instructions like “now it's blue, Berry Blue is winning so we move the joystick this way, see?” Then the experimenter let the children guide the joystick themselves commenting on who was “winning” and asking questions such as “who is winning now” to check their understanding. During the training phase, children were encouraged to recognize and report periods of mixed rivalry. Only children who clearly understood the task by the end of second repetition of the training phase continued to the test phase. All children passed this criterion, while one typical child and three autistic children required a second repetition of the training phase. These numbers were comparable between the two groups, *χ* ^2^(1, *N* = 50) = 1.35, *P* = 0.25. The mean number of training blocks was 1.19 ± 0.40 for autistic and 1.05 ± 0.22 for typical children.

#### Test phase

The test phase comprised four 120‐sec experimental sessions, two for each visual stimulus (gratings or house/face). Gratings and house/face stimuli were tested in two separate and consecutive blocks after one or more training sessions. The experimenter initiated each session with a button press, after confirming with the participant she/he was ready. The visual stimuli associated with each eye were swapped at every session, to reduce the possibility of response bias in favor of one or the other stimulus or character. The order of the gratings and house/face sessions was counterbalanced across participants.

### Analyses

Joystick responses were recorded every 10 ms. Joystick positions on the left‐right axis were a continuous signal ranging from 0, corresponding to the leftmost position, to 65,535, corresponding to the rightmost position (position 32,767 thus corresponded to the middle). We converted the continuous responses of the joystick into three discrete perceptual states (red, blue and mixed) using the following criterion: joystick positions within ±15% from the middle position were considered as mixed percepts; joystick positions greater than the middle position (+15%) were considered as blue percepts; and positions less than middle position (−15%) were considered as red percepts. Periods of mixed percepts shorter than 500 ms were excluded from the analyses, as they likely reflected artefacts due to the transition from one coherent percept to the other (to report a switch from one stimulus to the other, participants had to mandatorily pass through the middle position). The overall time periods removed were comparable across groups: the average time removed (across four 120‐sec trials, mean ± s.e.m.) for the autistic group was 706 ± 39 ms, 710 ± 41 ms for the typical group, *t*(34) = 0.08, *P* = 0.93. All subsequent statistical analyses were performed after the removal these periods. The first response point was considered when the child first moved the joystick in a trial (that is, the period prior to this time point was not taken as a mixed percept period).

We assessed rivalry dynamics by calculating several measures for each participant. A key measure was the number of perceptual transitions. Perceptual transitions included *real transitions* (participants switching from one visual stimulus to the other, sometimes with a period of mixed perception in between) and *reversions* (participants moving to a mixed phase and then switching back to the visual stimulus that was dominant before). To assess participants' performance taking into account this distinction, we also calculated the number and the proportion of reversions within the number of perceptual transitions.

To further characterize binocular rivalry dynamics, we measured the average durations when children reported perceiving one of the two stimuli (mean phase durations) and the average durations when they reported that they perceived mixed perception stimuli (duration of mixed percepts). Since one peculiarity of binocular rivalry is a nonsymmetrical distribution of phase durations (Levelt, 1966), we did not exclude outlier phase durations from the analyses. We also calculated the overall proportion of experimental time in which children reported exclusive perception of one of the two rivalrous stimuli, as well as mixed percepts. Finally, we measured the total number of reported red, blue and mixed percepts for each stimulus condition and each group.

We analyzed the two 120‐sec experimental sessions in which the same stimuli were presented (gratings or house/face) together. The so‐obtained measures were compared using 2 (within‐participants factor: stimulus type) x 2 (between‐participants factor: group) ANOVAs and *t*‐tests. We also examined correlations between these measures and background variables, including age and scores on the WASI‐II [Wechsler, 2011], SCQ (Rutter et al., 2003) and ADOS‐2 [Lord et al., 2012].

## Results

### Data Screening

The histogram of obtained raw responses shows clear peaks for the three relevant positions (right, left, middle) for both typical (Fig. 1E) and autistic (Fig. 1F) children. This suggests that the children in the two groups learned correctly how to report periods of red, blue and mixed percepts. The two groups also showed comparable latencies in their first responses (mean ± SD: typical children = 2.17 ± 1.2 sec; autistic children = 1.8 ± 1.3 sec, *F*(1,34) = 3.38, *P* = 0.08, *η*_p_^2^ = 0.09).

### Comparable Number of Transitions, Number of Reversions, and Proportion of Reversions in Autistic and Typical Children

**Figure 2:**
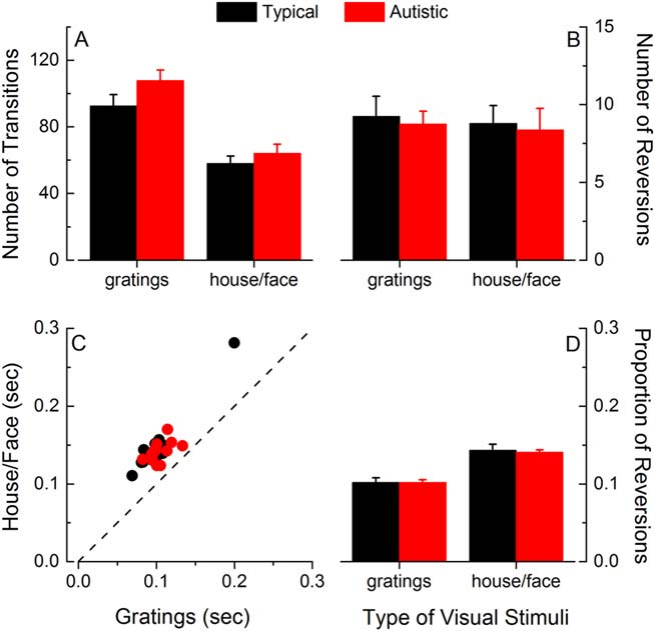
Number of perceptual transitions, reversions and proportion of reversions. (A): Average number of perceptual alternations in the 240‐sec experimental block. (B) Average number of perceptual reversions in the 240‐sec experimental block. (C, D): individual data (C) and average (D) proportion of reversions (ratio between the total number of reversions and the total number of perceptual transitions) for gratings and house/face stimuli in the two groups of children (black symbols: typical children, red symbols: autistic children). Error bars represent ±1 SEM.

A key index of binocular rivalry is the number of perceptual transitions (proportional to transition/alternation rate), which is shown in Figure 2A for the two groups of children and the two types of stimulus. Visual inspection suggests a trend for a higher number of perceptual transitions in the group of autistic children compared to the typical children [that is, in the opposite direction to Freyberg et al., 2015 and Robertson et al., 2013, 2016]. However, statistical analysis showed that this difference between the two groups was not significant, *F*(1,34) = 2.08, *P* = 0.16, *η*_p_^2^ = 0.06. The number of perceptual transitions was also lower for house/face stimuli compared to gratings, *F*(1,34) = 119.16, *P* < 0.0001, *η*_p_^2^ = 0.78. The group by stimulus type interaction was not significant, *F*(1,34) = 1.65, *P* = 0.21, *η*_p_^2^ = 0.05, suggesting that the number of perceptual transitions was affected by stimulus type similarly in the two groups.

Figure 2B shows the number of reversions, a special type of transition, in which a dominance phase of one of the two visual stimuli was interrupted by a period of mixed perception. The number of reversions did not differ between the two groups, *F*(1,34) = 0.1, *P* = 0.75, *η*_p_^2^ = 0.03, and no differences were observed in this measure for the two types of visual stimuli (*F*(1,34) = 0.21, *P* = 0.65, *η*_p_^2^ = 0.06). The ratio of *number of reversions: number of transitions* (Fig. 2C, D) was also considered to quantify the prevalence of reversions within transitions. This ratio was comparable in autistic and typical children, *F*(1,34) = 0.03, *P* = 0.86, *η*_p_^2^ = 0.001, suggesting that transitions in the two groups were qualitatively similar (same proportion of real transitions and reversions). Within each group, the proportion of reversions was larger for house/face stimuli compared to gratings, *F*(1,34) = 379.03, *P* < 0.0001, *η*_p_^2^ = 0.92, a pattern that was consistent across groups as the group x stimuli type interaction was not significant, *F*(1,34) = 0.5, *P* < 0.48, *η*_p_^2^ = 0.015.

### Comparable Mean Phase Durations and Mixed Percepts Durations for Autistic Children

Rivalry dynamics can still be different even if two participants present the same number of perceptual transitions and the same number of reversions if they perceive dominant and mixed percepts in different proportions. For example, one participant might spend more time reporting she/he perceives one of the two visual stimuli (holding the joystick in the left or the right position), while another participant might report mixed percepts for longer times (holding the joystick in the middle position). We addressed these aspects of rivalry dynamics in our data by examining mean phase durations and mixed percept durations.

**Figure 3:**
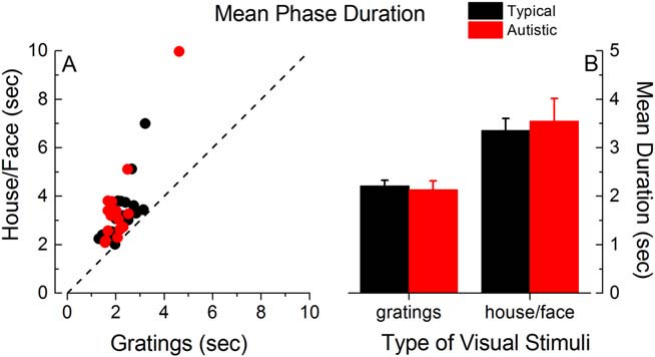
Binocular rivalry mean phase durations (average times spent by children reporting exclusive perception of one or the other visual stimulus) for gratings and for house/face stimuli. (A): Individual data, (B) group averages (black: typical children, red: autistic children). Error bars represent ±1 SEM.

Mean phase durations, defined as the average time of reported dominance of either of the rivalrous visual stimuli, (Fig. 3) did not differ between autistic and typical children, *F*(1,34) = 0.03, *P* = 0.87, *η*_p_^2^ = 0.001, a result which is consistent with the findings of Freyberg et al.'s [2015] adult study. However, mean phase durations differed across stimuli, being longer for house/face stimuli compared to gratings (main effect of stimulus type, *F*(1,34) = 53.48, *P* < 0.001, *η*_p_^2^ = 0.61. The group x stimuli interaction, however, was not significant, *F*(1,34) = 0.58, *P* = 0.45, *η*_p_^2^ = 0.02. The number of reported red and blue periods was also comparable between groups: blue, *F*(1,34) = 2.70, *P* = 0.11, *η*_p_^2^ = 0.07, red, *F*(1,34) = 0.73, *P* = 0.40, *η*_p_^2^ = 0.02, indicating that there was no effect of color.

**Figure 4:**
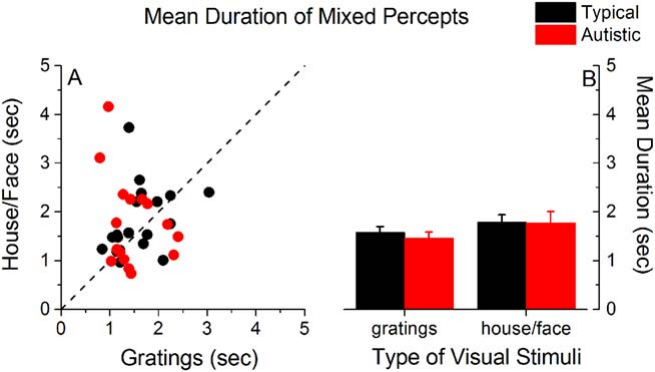
Mean duration of reported periods of mixed perception for gratings and house/face stimuli in the two groups of children. (A): Individual data; (B): group averages; black: typical children, red: autistic children. Error bars represent ±1 SEM.

Turning to the mean duration of mixed percepts (Fig. 4), our analysis revealed no significant group differences in the mean duration of mixed percepts (Fig. 4), *F*(1,34) = 0.15, *P* = 0.71, *η*_p_^2^ = 0.004. Moreover, within each group of children, mixed percepts were comparable for house/face and gratings stimuli, *F*(1,34) = 2.75, *P* = 0.11, *η*_p_^2^ = 0.07, while there was no significant group by stimulus‐type interaction, *F*(1,34) = 0.11, *P* = 0.74, *η*_p_^2^ = 0.003.

### Lower Proportion and Number of Mixed Periods in Autistic Children

**Figure 5:**
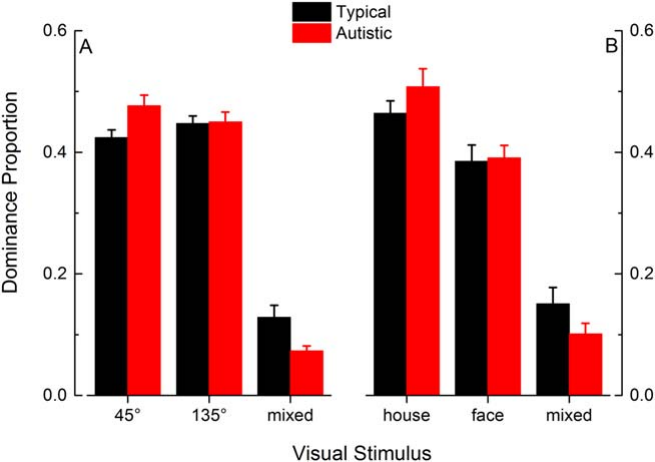
Dominance proportion, that is, the proportion of time in which children reported they perceived one of the two rivalrous stimuli, or a mixture of the two. (A): gratings; (B): house/face stimuli. Typical children are shown with black bars and autistic children with red bars. Error bars represent ±1 SEM and stars indicate significant between‐group differences.

We also examined mixed perception measuring the proportion of mixed perception (total time of reported mixed perception/240 sec viewing time, shown in Fig. 5) across experimental time, as well as the number of mixed perception periods (mean ± SD: typical children = 16.8 ± 8.6; autistic children = 11.5 ± 5.8). Autistic children showed a lower proportion of mixed perception and fewer mixed periods than typical children. For these two measures, however, between‐group differences reached statistical significance [proportion of mixed perception: *F*(1,34) = 4.18, *P* = 0.049, *η*_p_^2^ = 0.11; number of mixed periods: *F*(1,34) = 4.37, *P* = 0.04, *η*_p_^2^ = 0.11]. There was no difference, however, in mixed perception proportion or the number of mixed periods between house/face and grating stimuli [proportion of mixed perceptions: *F*(1,34) = 3.36, *P* = 0.76, *η*_p_^2^ = 0.09; number of mixed periods: *F*(1,34) = 0.69, *P* = 0.41, *η*_p_^2^ = 0.02] and no significant group by stimuli type interactions (ps > 0.72), *F*(1,34) = 0.04, *P* = 0.84, *η*_p_^2^ = 0.001. These results indicate that, even though autistic children showed comparable durations of mixed percepts compared to typical children, they experienced fewer epochs of mixed rivalry, a result that is reflected in the lower number and smaller proportion of mixed periods. This result indicates that mixed perception differed in autistic and typical children in a pattern that is opposite to the findings of Freyberg et al. [2015] and Robertson et al. [2013].

### Unconscious Preference for House Over Face, Especially for Autistic Children

To investigate whether participants showed a preference for one of the two rivalrous visual stimuli, we computed the proportion (total time of reported stimulus dominance/240 sec viewing time) of clockwise/anti‐clockwise gratings (Fig. 5A) and house/face (Fig. 5B) perception. We found no preference for either grating stimulus in autistic, *t*(15) = 0.82, *P* = 0.43, or typical children, *t*(19) = 1.5, *P* = 0.15. However, there was a trend for a preference for the house over the face stimulus in both groups of children, which was significant for autistic children, *t*(15) = 2.45, *P* = 0.03, but not for typically developing children, *t*(19) = 1.99, *P* = 0.06.

### Significant Links With Autistic Symptomatology, but Not With Age or Cognitive Ability

We also performed correlations between the four indices of binocular rivalry (transitions rate, mean phase duration, mean mixed percepts duration, proportion of mixed percepts) with children's age, SCQ and full‐scale IQ scores within each group, and with ADOS‐2 scores for autistic children only.

**Figure 6:**
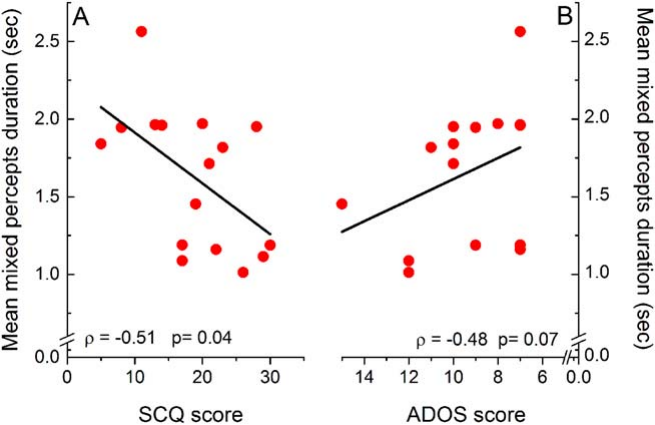
Correlations between mean duration of mixed percepts and autistic symptomatology scores. (A): SCQ [Rutter et al., 2003]; (B): ADOS‐2 (Lord et al., 2012).

Interestingly, this analysis showed a significant negative correlation between SCQ scores and the duration of mixed periods in autistic children (Fig. 6A, Spearman's rho = −0.51, *P* = 0.04, 95% CI = [0.02, 0.80]), as well as a nonsignificant trend for a negative correlation between the duration of mixed periods and the ADOS‐2 scores (Fig. 6B, Spearman's rho = −0.48, *P* = 0.07, 95% CI = [−0.79, 0.02]). These results, which indicated shorter periods of mixed perception in participants with more pronounced autistic symptoms, are consistent with the between‐group differences in measures related to mixed perception and are, again, opposite to the findings of Robertson et al. [2013] with autistic adults. There was, however, no correlation between autistic symptomatology and the proportion of mixed percepts.

No other correlations were significant (Spearman's rhos ranging from 0.02 to 0.39, *P*‐values ranging from 0.13 to 0.94), suggesting that there was no systematic relationship between rivalry dynamics and age or cognitive ability.

## Discussion

Many models of binocular rivalry suggest that reciprocal inhibition between neuronal populations representing the two rivalrous images is one of the main mechanisms underlying visual competition during binocular rivalry [Blake et al., 1998; Freeman, 2005; Lehky, 1988; Noest et al., 2007; Wilson, 2003]. These models, corroborated by evidence from MR Spectroscopy studies linking rivalry dynamics to the levels of GABAergic inhibition in the primary visual cortex [Lunghi et al., 2015; van Loon et al., 2013], would predict that in autism, which is thought to present an atypical E/I ratio [Rubenstein & Merzenich, 2003, Vattikuti & Chow, 2010], rivalry dynamics should be altered. In this study, we compared binocular rivalry dynamics in autistic and typical children of similar age and ability. Our paradigm also included different types of visual stimuli, to allow for the consideration of potential interactions between rivalry dynamics with stimulus complexity, semantic interpretation or social relevance.

Our analysis revealed no significant differences between the two groups of children in the number of transitions (∼ alternation rate) and the number and proportion of reversions (mixed perception followed by switching back to the previously dominant percept) within perceptual transitions. These findings suggested that, unlike autistic and neurotypical adults [Freyberg et al., 2015; Robertson et al., 2013, 2016], autistic and neurotypical children show similarities in the degree and nature (proportion of reversals) of transitions in binocular rivalry.

Interestingly, the picture changed when we focused on measures of the perception of one of the two stimuli and the perception of an amalgamation of the two stimuli (mixed‐percept duration). Mean phase durations (durations of perception of one of the two stimuli) were found to be highly similar in autistic and typical children, similar to results in the adult studies of Freyberg et al. [2015] and Robertson et al. [2013]. Mixed‐percept durations were also comparable, on average, in the two groups. Yet, the autistic children presented an interesting pattern of individual variability according to which children who showed greater degrees of autistic symptomatology demonstrated shorter mixed periods. The group of autistic children also presented a significantly lower proportion of mixed perception across experimental time and significantly fewer mixed periods compared to typical children. These latter results are in direct contrast to the pattern in the earlier adult studies [Freyberg et al., 2015; Robertson et al., 2013, as well as a trend in Said et al., 2013], which reported higher proportions of mixed perception in autistic adults compared to neurotypical adults.

Like the previous adult studies of rivalry [Freyberg et al., 2015; Robertson et al., 2013, 2016, but not Said et al., 2013], our findings support the idea that binocular rivalry is different in autism, in line with the predictions of the E/I hypothesis [Rubenstein & Merzenich, 2003; Vattikuti & Chow, 2010]. However, the dissimilarities between autistic and neurotypical children are qualitatively different to adults in two ways. First, differences are manifested mainly in measures related to mixed perception (rather than in transition rates). Second, the observed differences in mixed‐perception are in the opposite direction (lower vs. higher proportion of mixed perception/numbers of mixed periods) to the adult data [Freyberg et al., 2015; Robertson et al., 2013].

The first discrepancy between our study and previous adult studies could be accounted for by considering that phases of mixed perception, in which differences in the activation between groups of neurons representing competing images are subtler, should be less stable than phases of exclusive perception. Any underlying atypicalities in cortical inhibition would therefore affect mixed‐perception phases to a greater extent than the phases of exclusive perception [a pattern which also held for Freyberg et al., 2015]. In that vein, the presence of group differences only in mixed‐perception in our results could imply subtler group differences in the E/I ratio [Rubenstein & Merzenich, 2003; Vattikuti & Chow, 2010] in children compared to adults.

The second discrepancy between our study and the earlier adult studies is more difficult to account for. At first glance, our finding that mixed‐perception proportions are *lower* in autistic versus typical children than *higher*, as in autistic versus neurotypical adults, could be taken to suggest that the autism‐typical development difference in terms of the E/I ratio has a different sign in childhood compared to adulthood. Such an account is in principle possible, but it is challenging to specify plausible neurodevelopmental mechanisms underlying its implementation. For example, when does the reversal of the E/I imbalance take place in the developing autistic brain and how does it affect brain function? Is there a developmental phase in which there is no E/I imbalance in the autistic brain?

Another possibility is that the pattern of individual variability in mixed‐percept durations in the autistic children (elevated autistic symptomatology associated with shorter mixed‐percept durations) is suggestive of a delay in the development of processes relevant to binocular rivalry in autistic children with more pronounced autistic symptoms. This is plausible, as studies that examined binocular rivalry in typical children [Hudak et al., 2011: 9 year olds; Kovács & Eisenberg, 2005: 5‐6 year olds] have reported elevated levels of binocular rivalry compared to adults [see also Ukai, Ando, & Kuze, 2003 for developmental changes in adulthood]. Hudak et al. [2011] had accounted for their results on the basis of greater and faster relative contributions of neural adaptation in children, evidenced by greater effects of cumulative history on rivalry dynamics in the children data. One important limitation of this account is that developmental differences in these studies were reported for dominance durations (increasing with age), as well as alternation rates (decreasing with age). Our study found no group or age differences on these measures despite considering a relatively wide age range (7–14 years), while the analysis of differences in the proportion of mixed perception across experimental time and the number of mixed perception periods yielded small‐to‐medium effect sizes (*η*_p_^2^ = 0.11, in both measures). Based on the pattern of individual variability in our sample, future studies on rivalry in autism could focus on individuals with more pronounced autistic symptomatology.

It is also possible that the differences in mixed perception between autistic and typical children are not related to cortical inhibition to the extent posited by the adult studies of rivalry [Freyberg et al., 2015; Robertson et al., 2013, 2016; Said et al., 2013]. For example, they might be due to altered top‐down control in autistic children, as suggested by many accounts of autistic perception [Happé & Frith, 2006; Mitchell, Mottron, Souliéres, & Ropar, 2010], including our own proposal [Pellicano & Burr, 2012] for attenuated prior knowledge within a Bayesian framework of perceptual inference. Attenuated top–down control should result in abnormal rivalry dynamics, in particular elevated levels of rivalry and quicker transitions between percepts, consistent with the differences observed in our data. This idea is akin to accounts of binocular rivalry suggesting that, similarly to other phenomena of multistable perception [e.g., ambiguous three‐dimensional figures/shapes: Mamassian & Landy, 1998; ambiguous motion displays: Hupé & Rubin, 2003], it can be explained in terms of the interpretation of ambiguous sensory information in light of prior knowledge, experience and intention [Leopold & Logothetis, 1999].

Interestingly, the discrepancy between our data and the earlier adult studies could be explained within the so‐called hierarchical models of binocular rivalry [Freeman, 2005; Lee & Blake, 2004; Leopold & Logothetis, 1996; Wilson, 2003], which include both top‐down influences and inhibition. It is likely that atypicalities in rivalry dynamics in autistic children reflect atypicalities in top–down control processes rather than in cortical inhibition. One reason for this might be that differences in the E/I rate between autistic and typical children are washed out by the elevated levels of neural noise in childhood [e.g., Manning, Tibber, Charman, Dakin, & Pellicano, 2015]. By contrast, the role of cortical inhibition might be more critical in adulthood, where the levels of sensory noise are not as high.

Finally, one should also consider the possibility that the lower proportion of mixed perception across experimental time and the fewer mixed‐perception periods in autistic children reflect decision‐making biases, for example their higher levels of intolerance of uncertainty [Boulter, Freeston, South, & Rodgers, 2014; Neil, Choque‐Olsen, & Pellicano, 2016; Wigham, Rodgers, South, McConachie, & Freeston, 2015]. This possibility would imply that there might be no atypicality in binocular rivalry per se in autistic children and no underlying difference in the E/I rate or top‐down control. The observed lower proportion of mixed‐perception and fewer mixed perception periods might just stem from their aversion to ambiguous situations. For example, the training phase provided a very concrete scenario to children for how mixed percept might look like, which should be different from children's experience of mixed stimuli induced by dichoptic presentation. It is possible that typical children were more inclined to tolerate this ambiguity than autistic children. Alternatively, latent associations between intolerance to uncertainty and sensory sensitivities, particularly in autism, might underlie differential performance of autistic children in the task [Neil et al., 2016]. However, a challenge for accounts based on intolerance to uncertainty is to explain why intolerance to uncertainty affects binocular rivalry in a different way in autistic adults than children.

Turning to the differences in rivalry for the two types of stimuli, we found longer mean phase durations and a lower number of transitions for the house/face stimuli (high‐complexity) compared to grating (low‐complexity) stimuli. This result is consistent with previous literature on adults [e.g., Alais, Van Boxtel, Parker, & Van Ee, 2010; Rogers, Rogers, & Tootle, 1977] and suggests that processes related to recognition and semantic interpretation slow down rivalry in high‐complexity stimuli. This result also supports models of rivalry that include top‐down influences [Freeman, 2005; Lee & Blake, 2004; Leopold & Logothetis, 1996; Wilson, 2003] in addition to inhibition. Our findings suggest that stimulus complexity affects rivalry dynamics in a similar way in children and adults, despite the processes underlying the recognition of low‐ and high‐complexity stimulus maturing at different rates. However, one limitation of our study is that we did not assess children's recognition abilities for the high‐ and low‐complexity stimuli. We were also unable to measure individual differences in perceptual or motor responses to stimuli while participants watched smooth transition between images (via “playback trials”) within the already‐tight time constraints of our developmentally‐appropriate task. Future studies should address these issues in greater detail by adopting research designs that combine measures of recognition and measures of rivalry. Larger samples might also be considered to allow for more statistical power than our study.

Importantly, the effects of stimulus type on most of the measures examined in this study were the same for autistic and typical children, as indicated by the absence of a significant interaction between group and stimuli type. An exception to this pattern was the magnitude of the preference of the two groups of children for the house over the face stimuli, which was greater for autistic children, possibly reflecting an unconscious preference for non‐social information, consistent with reports of difficulties in the processing of socially‐relevant information [Uljarevic & Hamilton, 2012]. Eye tracking methods would be useful to examine whether increased preference for nonsocially relevant stimuli might occurs whilst looking patterns are superficially identical [cf. Fletcher‐Watson, Leekam, Benson, Frank, & Findlay, 2009]. If this were the case, binocular rivalry dynamics would be useful for indexing underlying atypicalities in the processing of socially‐relevant stimuli in autistic individuals.

In conclusion, our results broadly suggest that rivalry dynamics are differentially affected in adult and developing autistic children, though there is also some indication for effects of individual differences in the perception of specific stimuli in rivalry dynamics.
